# Cord blood antibodies following BBIBP‐CorV (Sinopharm) vaccination during pregnancy

**DOI:** 10.1002/iid3.874

**Published:** 2023-06-14

**Authors:** Sedigheh Hantoushzadeh, Nasim Eshraghi, Sarang Younesi, Mohammadreza Salehi, Nima Rezaei, Mohammad Mehdi Hasheminejad, Pegah Rashidian, Saeedeh Shirdel, Fatemeh Asadi, Marjan Ghaemi

**Affiliations:** ^1^ Vali‐E‐Asr Reproductive Health Research Center, Family Health Research Institute Tehran University of Medical Sciences Tehran Iran; ^2^ Nilou Laboratory Prenatal Screening Department Tehran Iran; ^3^ Department of Infectious Diseases Tehran University of Medical Sciences Tehran Iran; ^4^ Department of Immunology, Research Center for Immunodeficiencies, Children's Medical Center Tehran University of Medical Sciences Tehran Iran

**Keywords:** antibody, cord blood, COVID‐19, Iran, maternal mortality, pregnancy, Sinopharm, vaccine

## Abstract

**Objective:**

This study aimed to evaluate the maternal and umbilical cord blood antibody levels, after COVID vaccination during pregnancy.

**Method:**

The women who received the COVID‐19 vaccine (Sinopharm) during pregnancy were included. Maternal and cord blood samples were tested to detect the severe acute respiratory syndrome coronavirus 2 receptor binding domain (RBD) specific antibodies. In addition, obstetric information and side effects after vaccination were gathered.

**Result:**

A total of 23 women were included. Eleven pregnant women took two doses and 12 cases received a single dose of the vaccine. No IgM antibody was detected in any maternal blood or cord blood samples. The RBD‐specific Immunoglobulin G (IgG) antibody was positive in mothers receiving 2 doses of the vaccine and their infants. But the antibody titers were under the positive cut‐off threshold for the other 12 women who were vaccinated with a single dose. Women who received both doses of vaccine had significantly higher IgG levels than a single dose of Sinopharm (*p* = .025). The same result was demonstrated in infants born to these mothers (*p* = .019).

**Conclusion:**

There was a significant correlation between maternal and neonatal IgG concentrations. Although, receiving both doses of the BBIBP‐CorV vaccine (not 1 dose) during pregnancy is highly beneficial for increasing humoral immunity for the mother and fetus.

## Graphical abstract

Results of maternal and cord blood samples to find severe acute respiratory syndrome coronavirus 2 receptor binding domain specific antibodies.

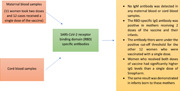

## 1 INTRODUCTION

Physiological changes during pregnancy may increase the risk of severe COVID‐19 disease. COVID‐19 infection can lead to many maternofetal morbidities, such as pre‐eclampsia, preterm birth, and intrauterine growth restriction.^1^, ^2^

Vaccination is a safe and effective public health measure to prevent severe COVID‐19 infection. But pregnant and lactating women were not included in primary COVID‐19 randomized clinical trials, so, at first, the vaccination during pregnancy was controversial.^3^, ^4^, ^5^ Based on a large number of recent studies on the nonpregnant population and increased risk of maternofetal complications with COVID‐19 disease, the American College of Obstetricians and Gynecologists and the Society for Maternal‐Fetal Medicine have recommended vaccination for all pregnant women.^6^, ^7^, ^8^, ^9^

In 2021, although the Sinopharm COVID‐19 vaccine (Vero Cell) had been approved under emergency use authorization by Food and Drug Administration, the safety and efficacy of vaccination during pregnancy had been unclear.^10^ In Iran, because of the high prevalence of COVID‐19 disease and many maternal mortalities caused by the coronavirus,^11^ the Iran ministry of health and medical education suggested that vaccination should be initiated in pregnant women.^12^ At first, only the Sinopharm vaccine was available for pregnant women in Iran. A recent study by Jeewandara et al. revealed that the seroconversion rate following Sinopharm/BBIBP‐CorV vaccination during the second and third trimesters of pregnancy is high, but they hadn't evaluated antibody concentration in cord blood. In addition, they demonstrated that getting a vaccine during pregnancy didn't lead to any adverse outcomes in pregnant women and their fetuses.^13^ Further, we attempted to evaluate the association between maternal and umbilical cord blood antibodies concentrations, after getting the Sinopharm vaccine during pregnancy.

## 2 METHOD

This is a preliminary study of pregnant women who vaccinated with at least one dose of inactivated BBIBP‐CorV vaccine (Sinopharm) during pregnancy and they admitted to a single medical center which is affiliated with the Tehran University of Medical Sciences (TUMS) for delivery from March to April 2021. Informed consent was obtained from all the participants included in this survey. Ethical approval for the present study was received from the institutional review board of TUMS. (No: IR.TUMS.MEDICINE.REC.1400.730).

Singleton pregnant women aged 16‐55 years old, who received one or two doses of the COVID‐19 vaccine (Sinopharm) during pregnancy (with more than the 21‐day interval between delivery and vaccination), were included in this study. The first dose should be administered between 13 and 36 weeks of gestation. It was suggested the second dose could be administered 3 weeks after the first dose. Pregnant women with multiple pregnancies, who were immunocompromised, and fetuses with severe congenital anomalies were excluded from the study.

All women were routinely tested for severe acute respiratory syndrome coronavirus 2 (SARS‐CoV‐2) infection, using polymerase chain reaction (PCR) from nasopharyngeal swabs at the time of admission for delivery, and the women with negative results were included. Demographics and obstetric data and also, the complications of vaccination were gathered for all participants. Maternal blood and umbilical venous blood samples were routinely collected for blood typing. Residual blood serums were obtained for performing laboratory studies.

To assess the humoral immune response, the SARS‐CoV‐2 receptor binding domain (RBD) specific antibodies (Immunoglobulin G [IgG] and IgM)were measured in both maternal and neonatal samples by using SARS‐CoV‐2 IgM and IgG ELISA kits.^14^ The cut‐off point for IgG and IgM ratios (the optical concentration of the sample/internal calibrator) in the samples was ≥1.1.

Analysis of data was performed by using SPSS (version 25.0, SPSS Inc.). Normal quantitative data was demonstrated by mean (standard deviation). Frequencies and percentages were used to present qualitative variables. The Pearson correlation was used to compare the antibody level among the maternal and neonatal blood samples and also, and it was used to evaluate the correlation between the time of the first dose administration of COVID‐19 vaccine during pregnancy and the cord blood antibody level. The significance level is *p* < .05.

## 3 RESULT

A total of 23 pregnant women were included in this study. Maternal and neonatal characteristics of pregnant women are demonstrated in Table 1.

**Table 1:** Maternal and newborn demographic and clinical data.

Characteristics	*n* = 23
Maternal age (year)	33.90 ( ± 4.72, 21−42)
BMI (kg/cm)	31.12 ( ± 5.01, 23−45)
Nulliparous	10 (43.4%)
Primigravid	9 (39.1%)
Current smoker	1 (4.3%)
Alcohol use	1 (4.3%)
Gestational diabetes mellitus	1 (4.3%)
Preterm labor (before 37 weeks)	1 (4.3%)
Neonatal birth weight	3287.55 ( ± 429.52)
Apgar score at 5 min < 7	0
Neonatal intensive care unit admission	1 (4.3%)
Umbilical arterial Ph. < 7.2	0

The side effects after getting the first and second doses of the Sinopharm vaccine are presented in Table 2. The most common complication was fatigue among women.

**Table 2:** Side effects of the vaccination after first and second dose.

	First dose of vaccine (*n* = 23)	Second dose of vaccine (*n* = 11)
Headache	1 (4.3%)	0
Chills	1 (4.3%)	0
Fever	1 (4.3%)	0
Temperature Above 38c	1 (4.3%)	0
Sweating	0	0
Decrease sleep quality	0	0
Palpitation	0	0
Heat or cold intolerance	1 (4.3%)	0
Injection site swelling or redness	1 (4.3%)	2 (8.6%)
Dizziness	0	0
Pain or discomfort in arm	1 (4.3%)	0
Nasal congestion	0	1 (4.3%)
Cough	0	0
Swelling of lips or oral cavity	0	1 (4.3%)
Allergic rhinitis	0	0
Myalgia	0	1 (4.3%)
Nausea or vomiting	1(4.3%)	0
Arthralgia	0	0
Fatigue	3 (13.0%)	1 (4.3%)
Anorexia	0	0
Euphoria	0	0
Flushing	0	0
Itching	0	0
Diarrhea	0	0
Somnolence	0	0
Heartburn	0	0
Seizure	0	0
Dyspnea	0	0
Hives	0	0
Anxiety	0	0
Atopic eczema	0	1 (4.3%)

Eleven pregnant women had two doses of the Sinopharm vaccine before the delivery and 12 women had only received a single dose vaccine. For all participants, the results of the PCR test for SARS‐CoV‐2 were negative when admitted for delivery they had no evidence of SARS‐CoV‐2 infection during pregnancy. The median time of the first vaccine dose administration was 29 (IQR 19.86−34.43) weeks of gestation.

No SARS‐CoV‐2 IgM antibody was detected in any of the maternal blood or cord blood samples. Women who received both doses of vaccine had significantly higher IgG levels 1.74 (IQR: 1.08−2.06) than mothers who were vaccinated with a single dose of Sinopharm 0.48 (IQR: 0.20−0.69) (*p* = .025). The same result was demonstrated in infants born to these mothers (*p* = .019) (Table 3).

**Table 3:** Immunoglobulin G (IgG) concentration in maternal and cord blood samples.

	Single dose vaccine	Two doses of vaccine	*p* Value
IgG level in maternal blood (median, IQR)	0.48 (0.20−0.69)	1.74 (1.08−2.06)	.025
IgG level in cord blood (median, IQR)	0.48 (0.20−0.66)	1.47 (0.82−2.58)	.019

The SARS‐CoV‐2 IgG was detected in 11 mothers and their infants who received two doses of the vaccine. But the antibody titers were under the positive cut‐off threshold for the other 12 women who were vaccinated with a single dose (median, IQR = 0.48, 0.20−0.69), and their neonates (median, IQR = 0.48, 0.2−0.66). Also, there was a significant strong association between the maternal SARS‐CoV‐2 IgG concentration and cord blood (*r* = 0.956, *p* < .001). The maternal‐to‐neonatal transfer ratio was defined as the concentration of IgG in cord blood divided by the maternal IgG level. The mean maternal‐to‐neonatal transfer ratio of SARS‐CoV‐2 IgG was 0.87 (IQR 0.76−1.17).

In the 11 pregnant women who had received the two doses of the Sinopharm vaccine, the median time interval from the first dose to delivery was 80 (IQR 65−95) days. Among the 11 fully vaccinated women, there was a significant positive correlation between the time since the first dose of vaccine and maternal serum IgG levels (*r* = .604, *p* = .049) and also a significant correlation with IgG concentration of neonatal blood samples (*r* = .658, *p* = .028). These correlations weren't significant in women vaccinated with a single dose.

## 4 DISCUSSION

This research was the first study that measured the SARS‐CoV‐2 RBD‐specific antibody titers in the matched blood samples of pregnant women and their newborns after getting the Sinopharm vaccine, in Iran. The results of the present study indicated that IgM antibody was not detected in any of the maternal or cord blood samples, that was consistent with a recent study.^15^ Also, it was showed that there was a significant correlation between maternal and neonatal IgG concentrations. In line with other studies, our findings confirmed that the administration of the COVID‐19 vaccine during pregnancy may lead to the transplacental transfer of antibodies from mother to fetuses and provide enough levels of antibodies in the neonates to potentially protect them against SARS‐Cov‐2 infection.^16^, ^17^, ^18^

Yang et al by assessing 1359 pregnant women, indicated that the administration of messenger RNA (mRNA) vaccine during pregnancy results in vaccine‐induced humoral immunity in pregnant women. Also, receiving a booster dose during the third trimester of pregnancy was related to greater anti‐spike IgG titers in maternal and cord blood.^19^ The evidence of another study revealed that receiving two doses of mRNA vaccine during pregnancy could reduce the rate of COVID‐19 hospitalization among infants during the first 6 months of life.^20^ In addition, the previous studies indicated that SARS‐CoV‐2 specific antibodies (IgG, IgM, and IgA) were transferred into the breast milk in women who were immunized with the mRNA vaccine, consequently, it can prevent COVID‐19 infection in infants.^8^, ^21^, ^22^, ^23^

Pregnancy would increase the susceptibility to severe infection and death from COVID‐19.^24^ Consistent with our results, the prior investigations strongly recommended that vaccination should be performed during pregnancy to provide maternal and infant protection against COVID‐19 disease.^22^, ^25^, ^26^ Therefore, it seems crucial to determine the optimum time of vaccination for pregnant women, to reach the highest level of maternal and neonatal immunization. Rottenstreich et al. presented that maternal vaccination early in the third trimester resulted in a greater maternal‐to‐neonatal transfer ratio of SARS‐CoV‐2 antibody and a higher neonatal immunoglobulin concentration, compared with vaccination in the late third trimester.^27^

The present survey demonstrated that the duration between getting the first dose of vaccine and delivery was significantly correlated to antibody levels in the maternal and cord blood samples. A recent prospective cohort survey performed by Bashi et al. have been demonstrated the same result.^28^ In addition, Rottenstreich et al. indicated that early vaccination in the third trimester, could increase disease protection in infants.^27^ Since the ratio of transplacental transfer IgG antibody was affected by the time of getting the first dose,^29^ further randomized controlled studies are needed to discover the ideal time for vaccine injection in pregnant women.

This research was one of the first studies that measured the SARS‐CoV‐2 antibody titers in the matched blood samples of pregnant women and their newborns, in Iran. The main limitations of our study included 1‐ the small sample size, 2—the majority number of women who received the first dose vaccine during the third trimester, and 3—the antibody concentration was measured in this study, but the subclasses of IgG were not determined. Although it was shown in previous studies, that COVID‐19 vaccine immunization is associated with antibody concentration in blood, other studies are required to determine the antibody level to effectively protect against COVID‐19 disease.^30^, ^31^

## AUTHOR CONTRIBUTIONS

**Sedigheh Hantoushzadeh and Sarang Younesi and Marjan Ghaemi**: Conceptualization. **Marjan Ghaemi and Fatemeh Asadi**: Review and editing. **Nasim Eshraghi and Mohammadreza Salehi and Nima Rezaei**: Writing—original draft. **Mohammad Mehdi Hasheminejad and Saeedeh Shirdel and Pegah Rashidian**: Methodology, software and analysis

## CONFLICT OF INTEREST STATEMENT

The authors declare no conflict of interest.

## Data Availability

The data is available upon request.
